# Supplementary material for: SMARCB1 missense mutants disrupt SWI/SNF complex stability and remodeling activity
Source: Nat Commun. 2026 Apr 8;17:4987. doi: 10.1038/s41467-026-71531-8 (PMC13237135; doi:10.1038/s41467-026-71531-8)
Supplement: Supplementary file 1 — Supplementary Information [file 41467_2026_71531_MOESM1_ESM.pdf]

Supplementary Information

SMARCB1 missense mutants disrupt SWI/SNF complex stability and remodeling activity

Garrett W Cooper[1,2], Benjamin P Lee[1,2], Won Jun Kim[3,4], Yongdong Su[1,2], Victor Z Chen[1,2], Eliseo Salas[5], Xiaoping Yang[4], Robert E Lintner[4], Frederica Piccioni[4], Andrew O Giacomelli[6], Thomas P Howard[3], Pritha Bagchi[7], Karen N Conneely[8], David E Root[4], Bo Liang[5], James C Gumbart[9], William C Hahn[3,4], David U Gorkin[10], Jaclyn A Biegel[11], Susan N Chi[3,12], Andrew L Hong[1,2,13**]

** Corresponding author: andrew.hong2@emory.edu

Author Affiliations:
[1] Department of Pediatrics, Emory University School of Medicine, Atlanta, GA, USA
[2] Aflac Cancer and Blood Disorders Center - Children’s Healthcare of Atlanta, Atlanta, GA, USA
[3] Dana-Farber Cancer Institute, Boston, MA, USA
[4] Broad Institute of MIT and Harvard, Cambridge, MA, USA
[5] Department of Biochemistry, Emory University School of Medicine, Atlanta, GA, USA
[6] Humber Polytechnic, Toronto, ON, Canada
[7] Emory Integrated Proteomics Core, Emory University, Atlanta, GA, USA
[8] Department of Human Genetics, Emory University School of Medicine, Atlanta, GA, USA
[9] School of Physics and School of Chemistry and Biochemistry, Georgia Institute of Technology, Atlanta, GA, USA
[10] Department of Biology, Emory University, Atlanta, GA, USA
[11] Department of Pathology, Children’s Hospital Los Angeles and Keck School of Medicine, University of Southern California, Los Angeles, California, USA
[12] Boston Children's Hospital, Boston, MA, USA
[13] Winship Cancer Institute, Atlanta, GA, USA

Supplementary Figure 1

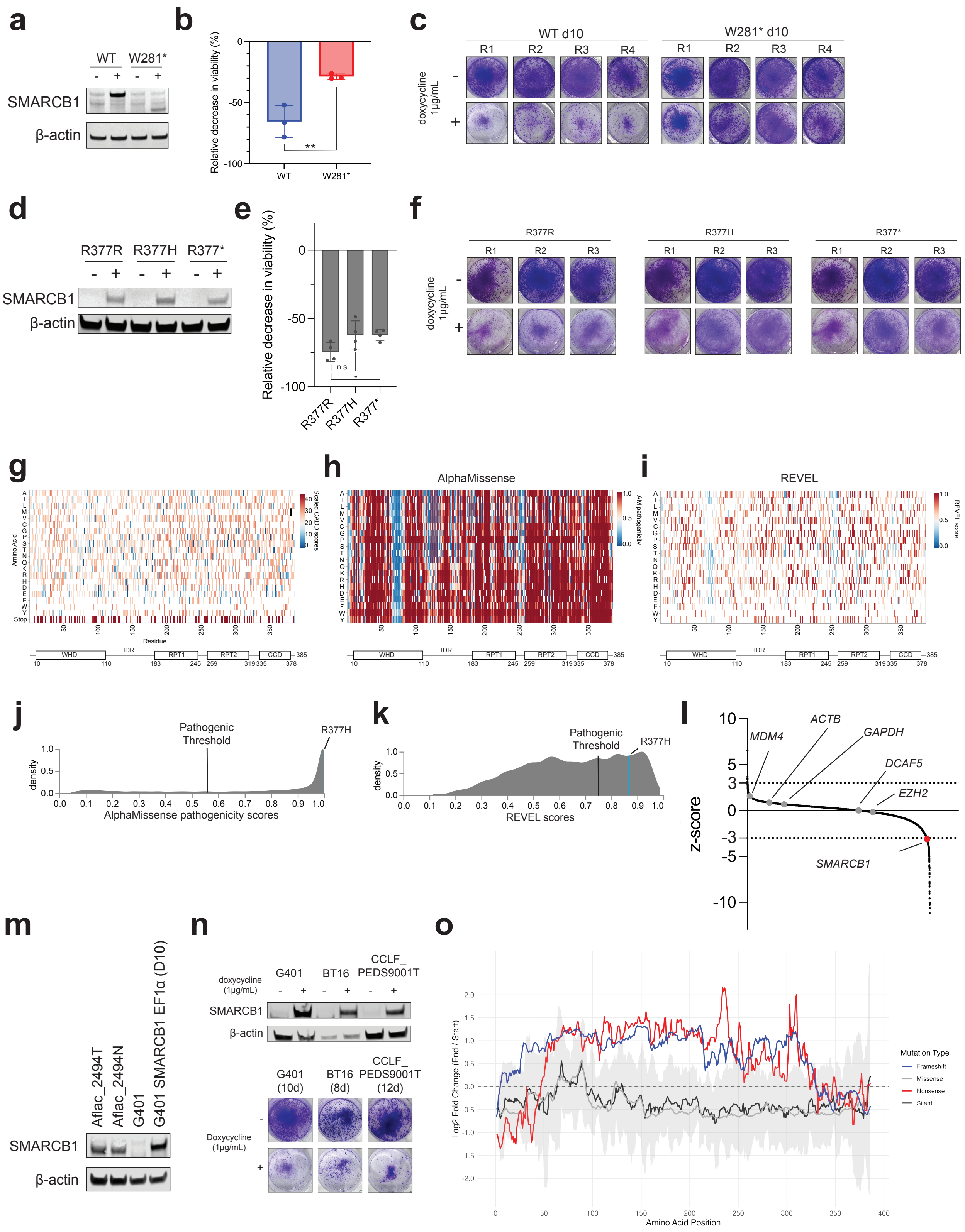

(a) Immunoblot showing inducible expression in the G401 cell line of wild type SMARCB1 and the patient-derived W281* nonsense mutation after 48 hours of induction with 1 μg/mL doxycycline.

(b) Cell counts as assessed by trypan blue exclusion after 8 days of induction. Wild type and W281* proliferation data also presented in Fig 2i and Supplementary Fig. 5e. Data are presented as mean values ± SD with individual data points overlaid (n=3 independent biological replicates, each from a separate passage). Statistical significance was assessed using two-tailed unpaired *t* tests (df=4). Exact *p*-values: WT v W281P: 0.0086, ***p* < 0.01. No adjustments were made for multiple comparisons.

(c) Crystal violet for both wild type and W281* induction after 10 days of induction (n=3). Crystal violet staining for wild type and W281* SMARCB1 is also presented in Fig 2d and Supplementary Fig 5a. Wild type crystal violet data is also presented in Supplementary Fig 1n.

(d) Immunoblot showing inducible expression in the G401 cell line of the silent R377R, missense R377H, and nonsense R377*, SMARCB1 constructs after 48 hours of induction with 1 μg/mL doxycycline.

(e) Cell counts as assessed by trypan blue exclusion after 8 days of induction. Data are presented as mean values ± SD with individual data points overlaid (n=4 independent biological replicates, each from a separate passage). Statistical significance was assessed using two-tailed unpaired *t* tests (df=6). Exact *p*-values: R377R v R377H: 0.0889; R377R v R377*: 0.0197; **p* < 0.05. No adjustments were made for multiple comparisons.

(f) Crystal violet for each construct (R377R, R377H, R377*) after 10 days of induction.

(g) Predicted pathogenicity of all predicted variants in the *SMARCB1* coding sequence from CADD v1.7.

(h) Predicted pathogenicity of all predicted variants in the *SMARCB1* coding sequence from AlphaMissense.

(i) Predicted pathogenicity of all predicted variants in the SMARCB1 coding sequence from REVEL v1.3

(j) Density plots of all missense mutations predicted from AlphaMissense. Pathogenic threshold applied at a pathogenicity score value of 0.56.

(k) Density plots of all missense mutations predicted from REVEL. Pathogenic threshold applied at a REVEL score value of 0.78.

(l) Genes from the hORFeome V8.1 Library ORF screen in G401 cell line ranked by z-score (*SMARCB1* highlighted in red, control genes such as *ACTB* and *GAPDH* highlighted in grey).

(m) Immunoblot of Wilms tumor patient derived cell line, adjacent normal kidney tissue, parental G401 cell line, and G401 cell line with constitutive SMARCB1 expression using an EF1α promoter for 10 days

(n) Immunoblot and crystal violet staining of SMARCB1 induction in G401, BT16, and CCLF_PEDS9001_T1 cell line. Wild type G401 crystal violet staining is also presented in Supplementary Fig 1c and Fig 1h.

(o) Raw log2FoldChange values for each variant type averaged across all three cell lines. Rolling minimum effect size range for a specific variant to be called significant (z-score > |2|) shown in gray.

Supplementary Figure 2

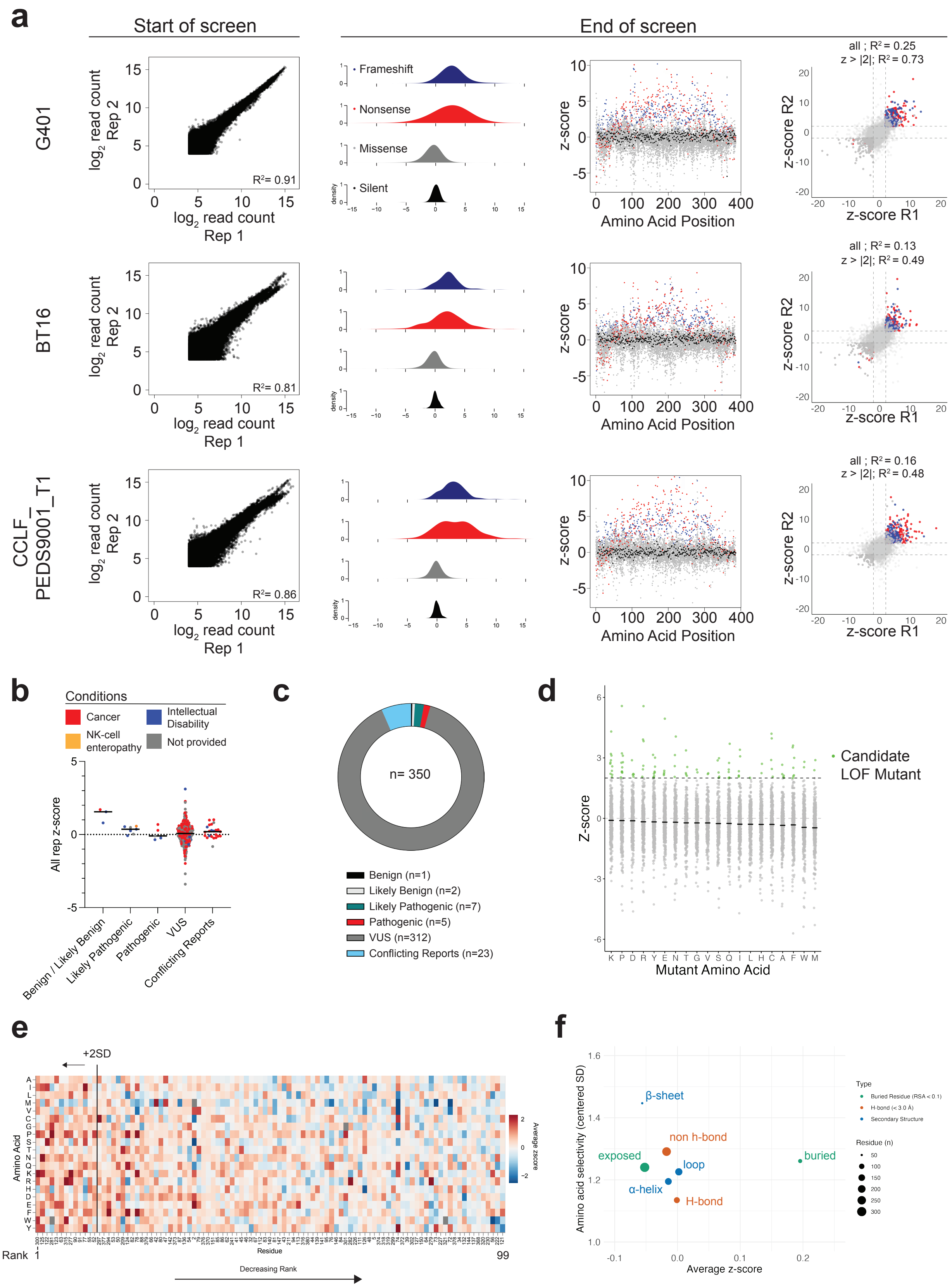

(a) Correlation of all mutant counts in the DMS library for each cell line at the starting time point after antibiotic selection. Density plots showing the functional z-score distribution for each cell line used across the 4 different types of mutations: frameshift, nonsense, missense, and silent. Z-score represent the average of two biological replicates. A functional z-score > 0 denotes mutation enrichment and < 0 denotes mutation depletion compared to silent mutations. Averaged functional z-score across each cell line for silent (in black), frameshift (in blue), and nonsense mutations (in red) across the length of the *SMARCB1* coding sequence. Correlation between two biological replicates for each cell line. Gray points represent nonsignificant variants; colored points highlight variants with |z-score| > 2 in both replicates. R^2 values are shown for all variants and for functionally significant variants separately.

(b) ClinVar predicted missense variants with their corresponding z-score colored by associated disease and grouped by variant classification.

(c) Circle plot depicting proportion of each category of 'Germline' classification as assessed through ClinVar.

(d) Distribution of functional z-scores by mutant amino acid identity. Z-scores represent the average across all three cell lines for each variant. Green points indicate functionally significant variants (z-score > 2); black horizontal line shows the mean z-score for each amino acid substitution.

(e) Heatmap showing z-scores for all amino acid substitutions at each residue position, ranked by mean functional effect (as in Fig 2d). Each column represents a residue; each row represents a substitution to a different amino acid. The black line denotes the threshold for residues with mean z-score > 0.679 (+2SD from the global mean).

(f) Amino acid selectivity versus mean functional effect for SMARCB1 residues grouped by structural features. Selectivity is measured as the centered standard deviation of z-scores across all amino acid substitutions at each position. Point size reflects the number of substitutions measured. Residues are categorized by: solvent accessibility (RSA < 0.1 = buried), hydrogen bonding (side chain interactions ≤ 3.0 Å), or secondary structure assignment from AlphaFold predictions.

Supplementary Figure 3

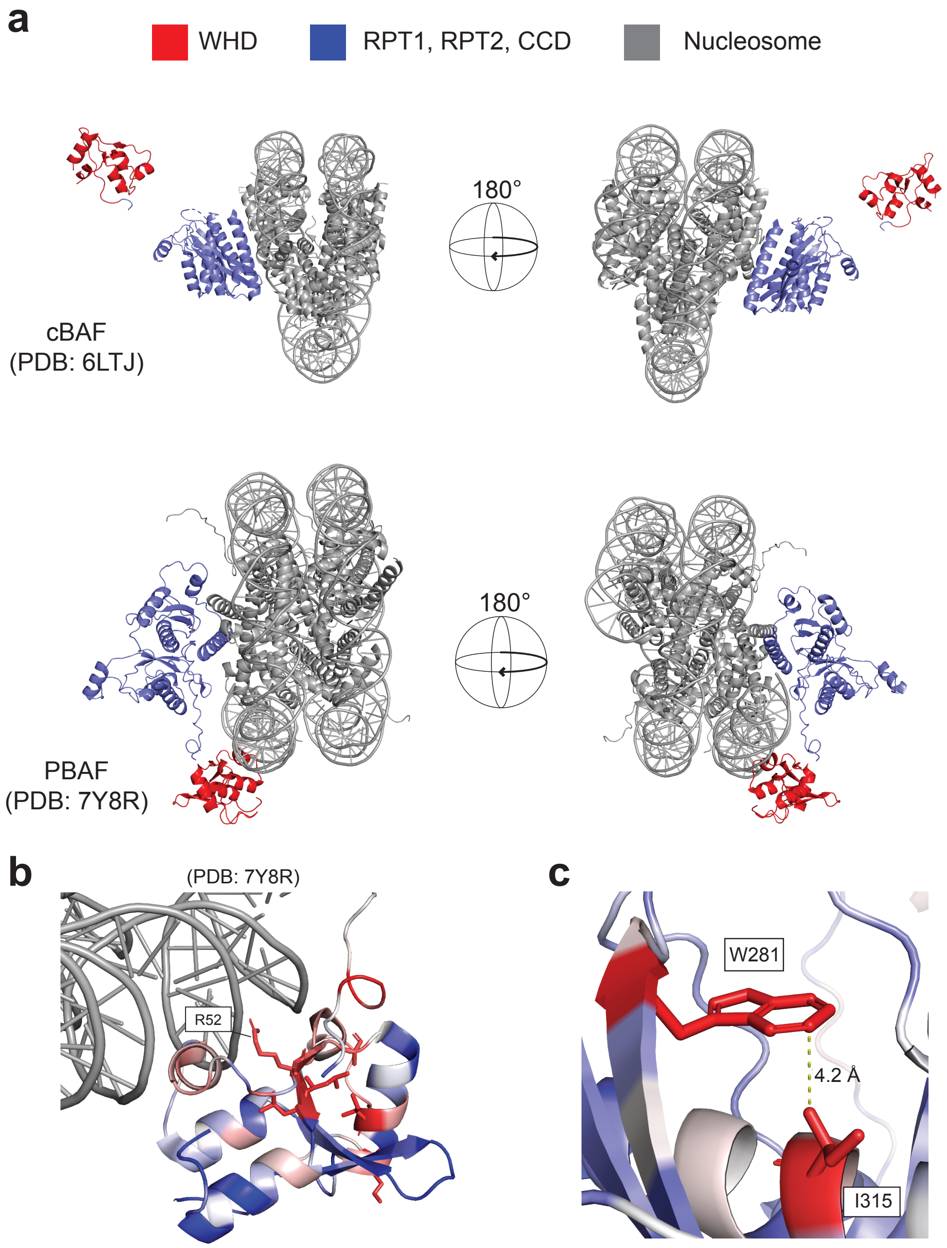

(a) Structural analysis of the two SMARCB1 containing SWI/SNF subfamilies, cBAF and PBAF, showing SMARCB1 is proximal to the DNA in PBAF and distal in cBAF.

(b) PBAF cryo-EM structure (PDB: 7Y8R) of WHD binding to DNA with residue averaged z-score overlaid. Intolerant residue R52 is labeled.

(c) Close interaction of residues W281 and I315R with residue averaged z-score overlaid on AlphaFold predicted structure of SMARCB1.

Supplementary Figure 4

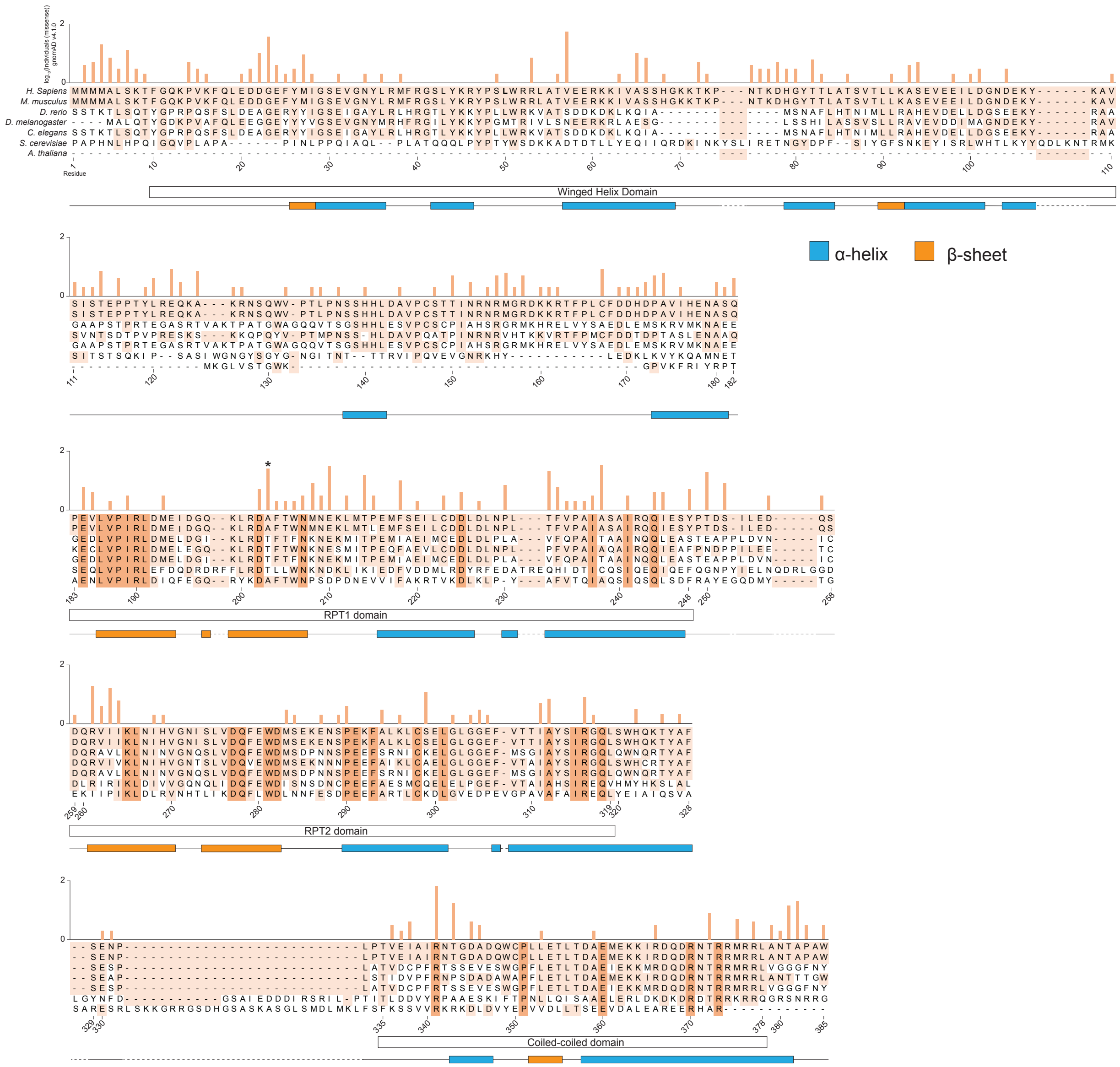

Multiple sequence analysis of SMARCB1 orthologs across seven eukaryotic species: *H. sapiens* (NP_003064.2), *M. musculus* (BAB12427.1), *D. rerio* (NP_001007297.1), *D. melanogaster* (AAC77830.1), *C. elegans* (NP_001369845.1), *S. cerevisiae* (ONH79494.1), and *A. thaliana* (NP_001189918). Orange shading indicates amino acid conservation. Lighter shading indicates residues with at least one species showing conservation with *H. sapiens*, and darker shading represents conservation across all seven species. Bar graph above the alignment shows the number of individuals with missense variants per residue from gnomAD v4.1.0 (all individuals were heterozygotes except one homozygous A203T individual denoted by *). Secondary structural elements (α-helix in blue, β-sheet in orange) are shown below based on AlphaFold structure predictions.

Supplementary Figure 5

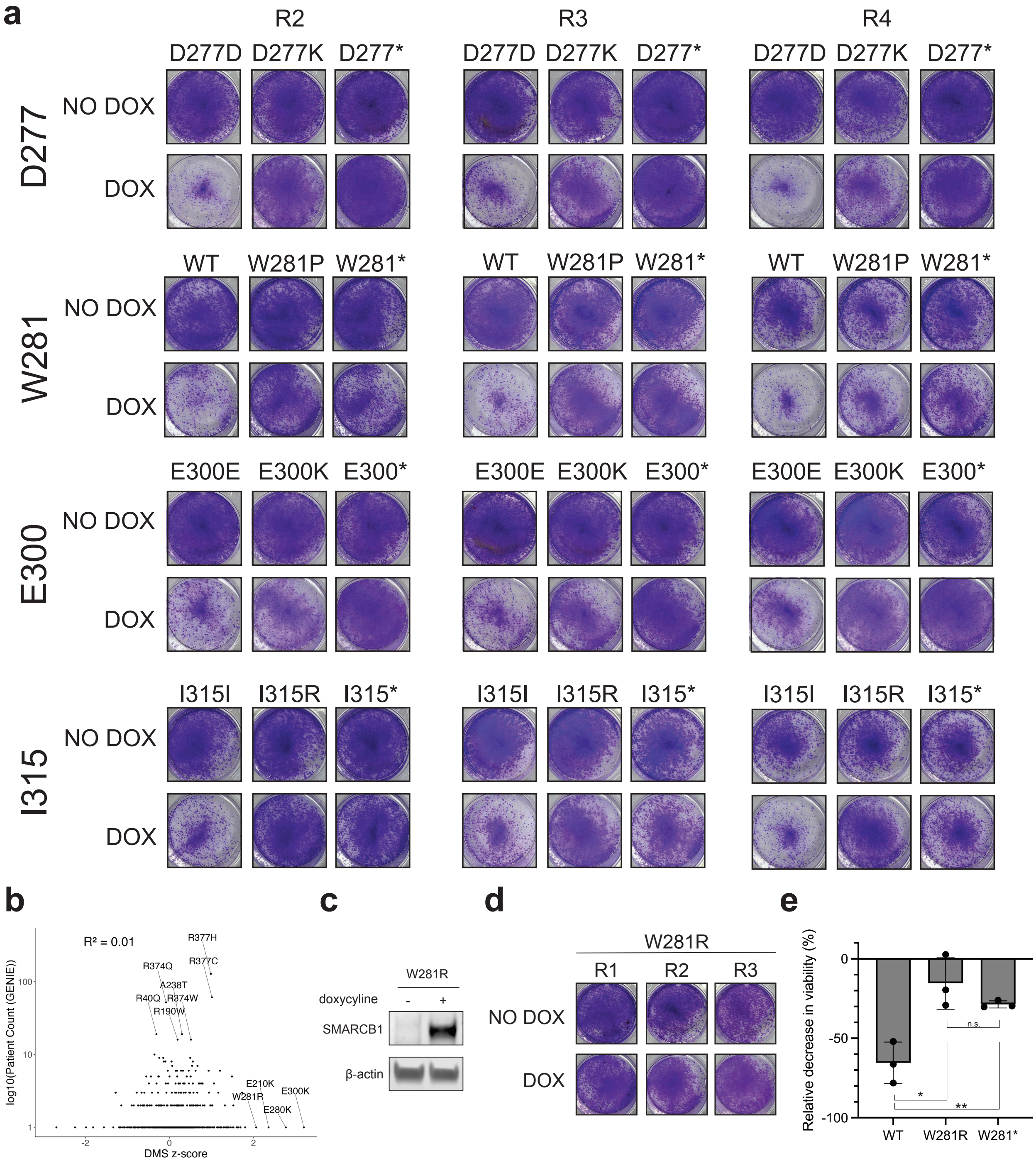

(a) Replicates (n=3, independent biological replicates from separate passages) of crystal violet staining of G401 cells after 10 days of induction for representative images shown in Fig 2h. Crystal violet staining of wild type and W281* also presented in Supplementary Fig. 1c.

(b) Comparison of functional effects (z-score) and cancer mutation frequency (GENIE). Functional z-scores (x-axis) are plotted against GENIE patient counts (y-axis, log10 scale) for all SMARCB1 missense variants. Variants with z-score > 2 or >10 patient observations are labeled.

(c) Immunoblot showing inducible expression from total protein lysates in the G401 cell line of patient-derived SMARCB1 missense mutant, W281R.

(d) Crystal violet staining of G401 cells after 10 days of induction of the W281R SMARCB1 mutant (n=3, independent biological replicates from separate passages).

(e) Cell counts as assessed by trypan blue exclusion after 8 days of induction in G401 cell line for the wild type, missense (W281R), and corresponding nonsense (W281*). Wild type and W281* data also presented in Supplementary Fig 1b and Fig 2i. Data are presented as mean values ± SD with individual data points overlaid (n=3 independent biological replicates, each from a separate passage). Statistical significance was assessed using two-tailed unpaired *t* tests (df=4). Exact *p*-values: WTvW281R: 0.0143; WTvW281*: 0.0086; W281PvW281*: 0.2348; $^*p < 0.05$, $^{**}p < 0.01$. No adjustments were made for multiple comparisons.

Supplementary Figure 6

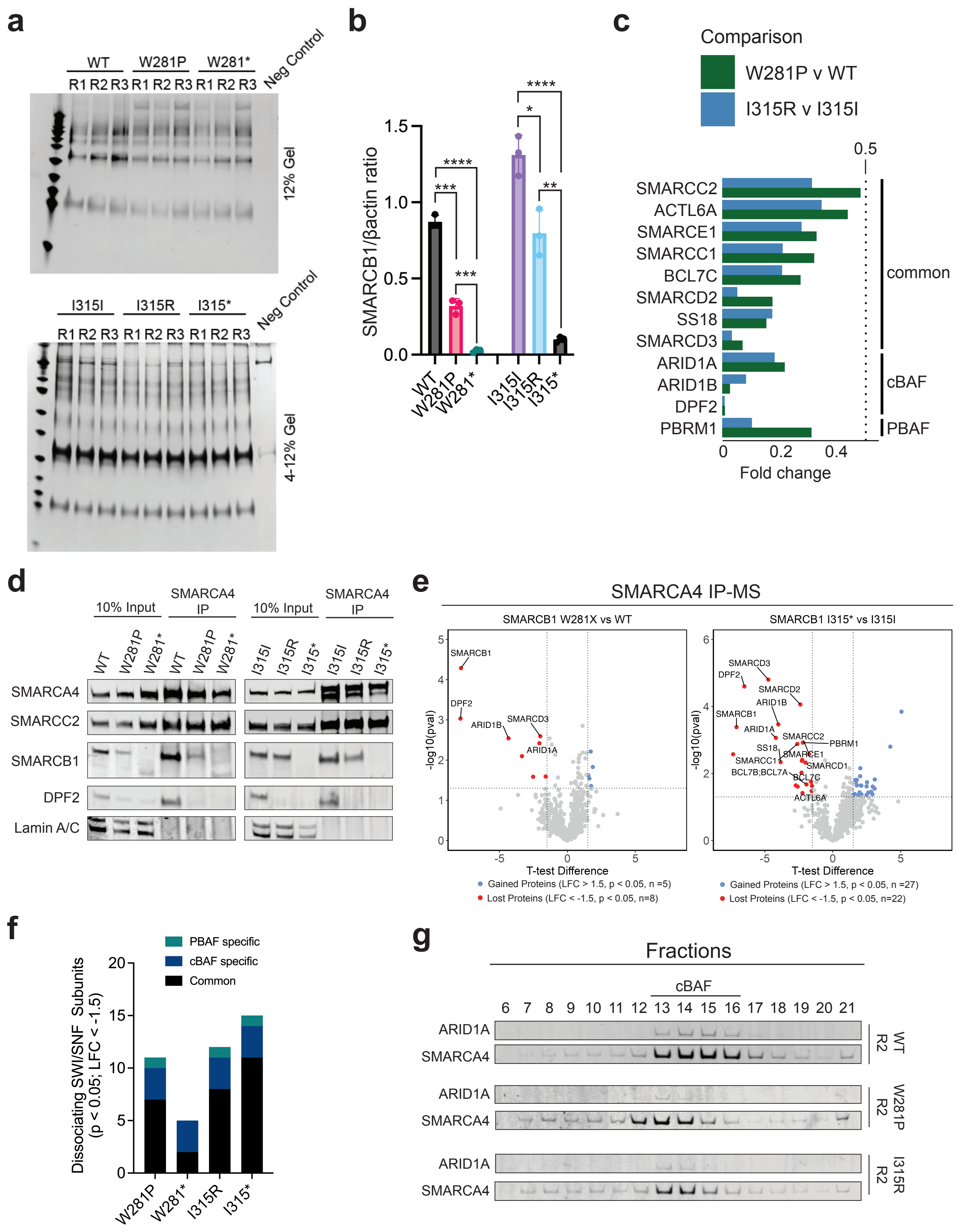

(a) Silver staining of mass spec inputs.

(b) Quantification of mass spec nuclear input shown in Fig 3a. *$p < 0.05$, **$p < 0.01$, ***$p < 0.001$, ****$p < 0.0001$ from a Student's two-tailed unpaired *t* test (df =4). Exact *p*-values: WT vs W281P: 0.0001; WT vs W281*: 4.9e-6; W281P vs W281*: 0.0005; I315I vs I315R: 0.0114; I315I vs I315*: 8.89e-5; I315R vs I315*: 0.0014. No adjustments were made for multiple comparisons.

(c) Fold change in protein abundance for SWI/SNF complex subunits in SMARCB1 mutant cell lines compared to wild type. Twelve subunits showing log2 fold change < -1.5 in at least one mutant (W281P or I315R/I315I) are displayed. Subunits are grouped by SWI/SNF complex type: common (present in all complexes), cBAF (canonical BAF-specific), or PBAF (polybromo-associated BAF-specific). Dashed line at 0.5 fold change indicates 50% reduction in protein levels associated with SMARCA4 in the mutant compared to wild type.

(d) Immunoblots of nuclear input and SMARCA4 IP showing complete dissociation of DPF2 in the W281P and I315R mutant conditions.

(e) Volcano plots of mass spectrometry data showing significant proteins (in red or blue) observed when comparing the nonsense mutants to the wild type constructs.

(f) Number of dissociated subunits in each comparison of mass spectrometry data. Dissociated subunits specific to either cBAf or PBAF are indicated in the legend.

(g) Replicate glycerol gradients for WT and missense mutant SMARCB1 constructs for those shown in Fig 3e.

Supplementary Figure 7

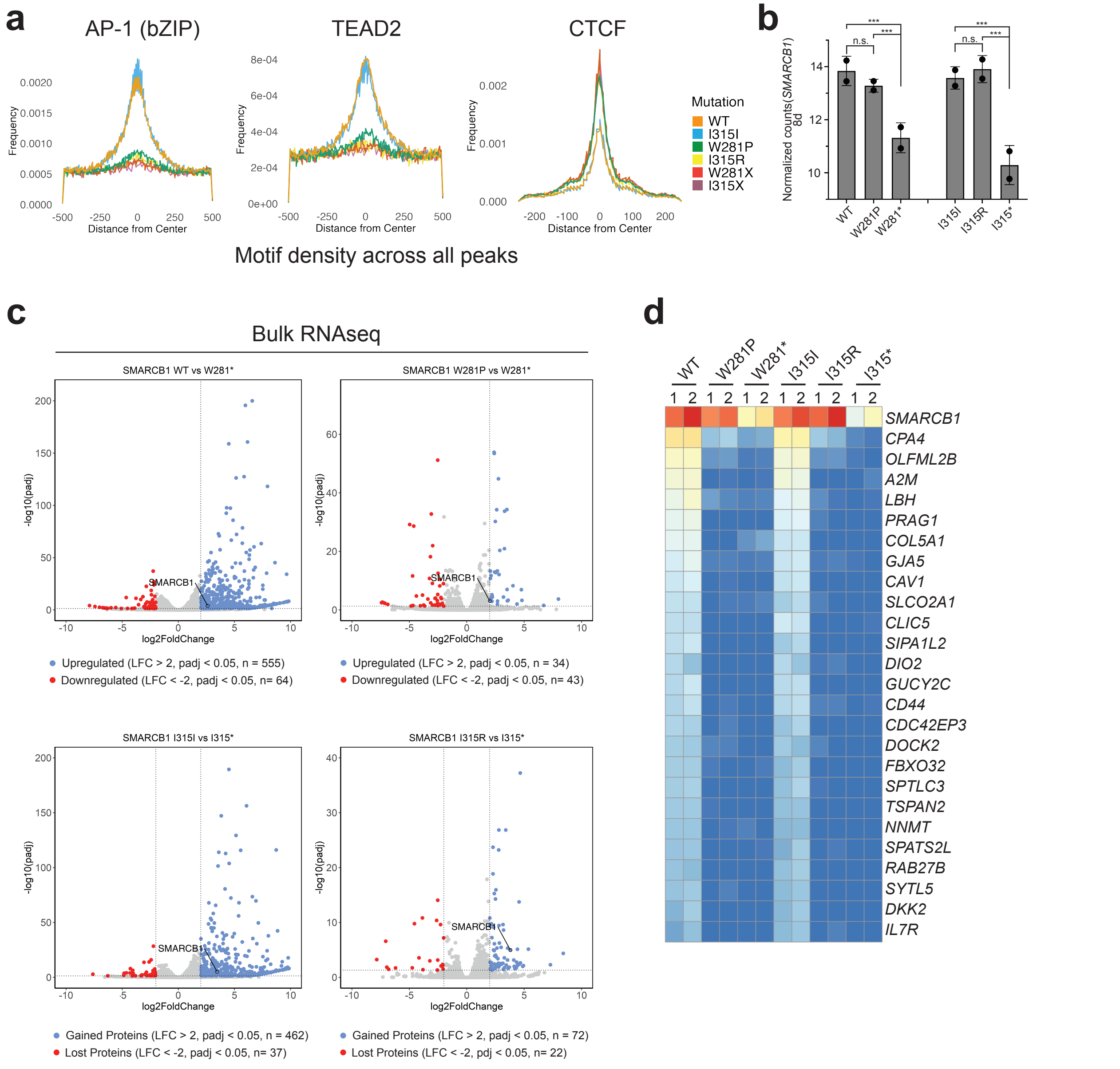

(a) Aggregate motif density profiles for AP-1 (bZIP family), TEAD2, and CTCF across ATAC-seq peaks in -type (WT, I315I) and mutant conditions (W281P, I315R, W281X, and I315X). Motif occurrences are plotted as a function of distance from peak centers (x-axis, in base pairs) with frequency shown on the y-axis.

(b) SMARCB1 transcript abundance for each condition as assessed through bulk RNA-seq after 8 days of induction with 1µg/mL doxycycline induction. ****padj* < 0.001 from differential expression analysis using DESeq2.

(c) Volcano plots of differentially expressed genes as assessed through DESeq2 found upon comparison of both wildtype and missense to its corresponding nonsense construct. Data summarized in Fig 4f.

(d) Normalized transcript abundance across all six conditions when looking at the top 25 most highly differentially expressed genes when comparing I315I to I315*.

Supplementary Immunoblots

Supplementary Figure 1a

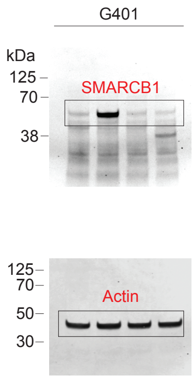

Supplementary Figure 1d

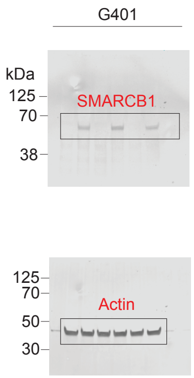

Supplementary Figure 1m

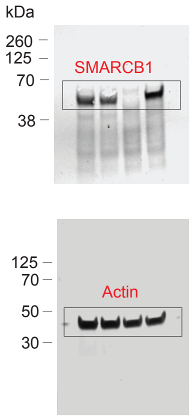

Supplementary Figure 1n

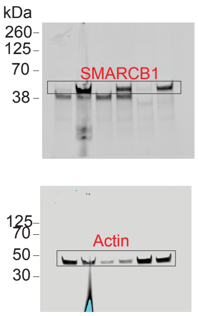

Supplementary Figure 5c

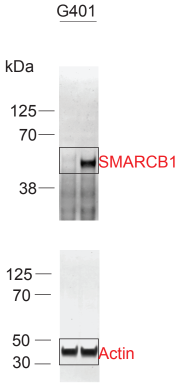

Supplementary Figure 6d

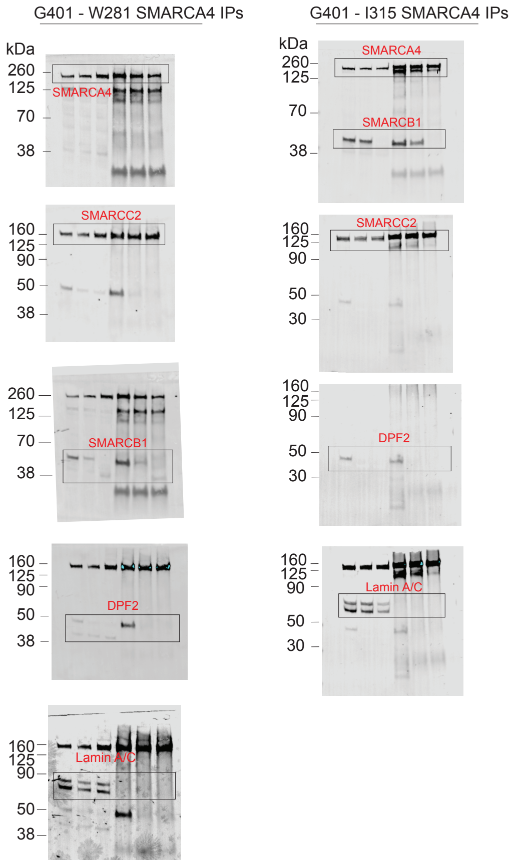

Supplementary Figure 6g

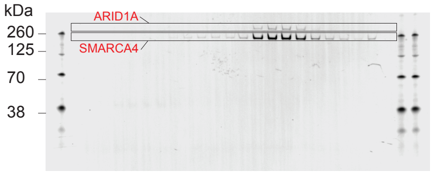

WT R2

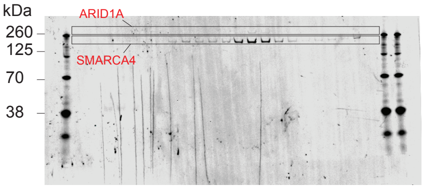

W281P R2

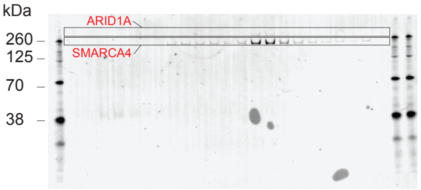

I315R R2
