# Supplementary material for: SMARCB1 missense mutants disrupt SWI/SNF complex stability and remodeling activity
Source: Nat Commun. 2026 Apr 8;17:4987. doi: 10.1038/s41467-026-71531-8 (PMC13237135; doi:10.1038/s41467-026-71531-8)
Supplement: Supplementary file 2 — Reporting Summary [file 41467_2026_71531_MOESM2_ESM.pdf]

nature portfolio

Corresponding author(s): Andrew L. Hong

Last updated by author(s): Feb 20, 2026

Reporting Summary

Nature Portfolio wishes to improve the reproducibility of the work that we publish. This form provides structure for consistency and transparency in reporting. For further information on Nature Portfolio policies, see our Editorial Policies and the Editorial Policy Checklist.

Statistics

For all statistical analyses, confirm that the following items are present in the figure legend, table legend, main text, or Methods section.

n/a	Confirmed	
☐	☒	The exact sample size (*n*) for each experimental group/condition, given as a discrete number and unit of measurement
☐	☒	A statement on whether measurements were taken from distinct samples or whether the same sample was measured repeatedly
☐	☒	The statistical test(s) used AND whether they are one- or two-sided<br>*Only common tests should be described solely by name; describe more complex techniques in the Methods section.*
☒	☐	A description of all covariates tested
☐	☒	A description of any assumptions or corrections, such as tests of normality and adjustment for multiple comparisons
☐	☒	A full description of the statistical parameters including central tendency (e.g. means) or other basic estimates (e.g. regression coefficient) AND variation (e.g. standard deviation) or associated estimates of uncertainty (e.g. confidence intervals)
☐	☒	For null hypothesis testing, the test statistic (e.g. *F*, *t*, *r*) with confidence intervals, effect sizes, degrees of freedom and *P* value noted<br>*Give P values as exact values whenever suitable.*
☒	☐	For Bayesian analysis, information on the choice of priors and Markov chain Monte Carlo settings
☒	☐	For hierarchical and complex designs, identification of the appropriate level for tests and full reporting of outcomes
☐	☒	Estimates of effect sizes (e.g. Cohen's *d*, Pearson's *r*), indicating how they were calculated

Our web collection on statistics for biologists contains articles on many of the points above.

Software and code

Policy information about availability of computer code

Data collection

Immunoblot chemiluminescent were acquired using the Odyssey Infared Imaging System (v3.0.30). Cell viability was measured through trypan blue exclusion using the Vi-Cell XR Cell Viability Analyzer. Sequencing reads for ATAC-seq experiments were obtained using the Illumina NextSeq 2000 to a depth of at least 50M paired-end reads. Sequencing reads for RNA-seq experiments were obtained using the Illumina NovaSeq 6000 to a depth of at least 20M paired-end reads.

LC-MS/MS data were collected using the Orbitrap Fusion Lumos Mass Spectrometer (ThermoFisher Scientific, San Jose, CA). Peptides were separated using a Water's Charged Surface Hybrid (CSH) column (150 µm internal diameter (ID) x 15 cm; particle size: 1.7 µm).

NGS processing tools:
Adaptor trimming, duplicate removal, and alignment for ATAC-seq analysis was performed using Illumina DRAGEN (v4.2.4). Alignment and gene quantification for RNA-seq analysis was performed using Illumina DRAGEN (v3.7.5). Subsequent analysis used: samtools (v1.19.2), bedtools (v2.31.1), MACS3 (v3.0.1), deepTools (v3.5.4), DiffBind (v3.5.4), HOMER (v4.11.1), DESeq2 (v1.46.0), ggplot2 (v3.5.1), pheatmap (1.0.12), dplyr (v1.1.4), RStudio (v2022.07.2), R (v4.4.2), IGV (2.14.1).

Molecular Dynamic Simulations:
Initial structures were obtained from AlphaFold v2.0 (DeepMind) and mutated using PyMol (v 2.5.4). MD simulations were performed on a Ubuntu operating system (v20.04) using a NVIDIA RTX A6000 graphics card running CUDA (12.6). MD simulations were perfomed using AmberTools(v24), trajectories were analyzed using VMD (v1.9.4) and ggplot2 (v3.5.1).

Proteomic processing tools:
MaxQaunt (v2.6.7), Perseus (v2.0.11.0), ggplot2 (v3.5.1), dplyr (v1.1.4).

Structural Modelling:
PyMol (v2.5.4), AlphaFold v2.0 (DeepMind)

Data analysis

The code for analyzing the data and the raw data tables have been deposited in GitHub (https://github.com/ahonglab/SMARCB1_DMS).

LC-MS/MS and Proteomics Analysis
Peptides were analyzed using LC-MS/MS with an Orbitrap Fusion Lumos Mass Spectrometer, and spectra were processed with MaxQuant (v2.6.7) for label-free quantification. Variable modifications included methionine oxidation, deamidation, and N-terminal acetylation, with a 1% FDR applied. Quantification was based on razor and unique peptides, and downstream statistical analysis was performed using Perseus (v2.0.11).

RNA Sequencing
RNA-seq libraries were sequenced using Illumina NovaSeq 6000 with at least 20M paired-end reads per sample. Data were aligned and quantifies using Illumina Dragen v3.7.5 to GrCh38p13. Differential gene expression analysis was conducted using DESeq2, with FDR < 0.05 and log2FC > |2| as significance thresholds.

ATAC Sequencing
ATAC-seq libraries were sequenced with a depth of 50M paired-end reads using Illumina NextSeq 2000. Reads were processed and aligned to GrCh38p13 with Dragen (v4.2.4), samtools, and bedtools, with quality filtering and exclusion of ENCODE blacklisted regions. Peaks were called with MACS3 with –nomodel -q 0.01 flag (high confidence peaks). Differential accessibility analysis was performed using DiffBind and DESeq2, with FDR < 0.05 and log2FC > |1| as cutoffs. Peaks were annotated with HOMER (v4.11.1), where each peak was assigned to the closest transcription start site (TSS) of a gene. Signal intensity for each significantly differential accessible peak was visualized using deepTools (v3.5.4). Motif enrichment was performed using HOMER (v4.11.1), where significantly differential peaks were analyzed against the hg38 reference genome using the findMotifsGenome.pl function. The analysis was conducted with default parameters and included a random background model to evaluate motif enrichment.

CUT&RUN Sequencing
CUT&RUN libraries were sequenced with a depth of 35M paired-end reads with at least 25M paired-end reads using the NovaSeq X Plus. Reads were processed and aligned to the GrCh38p13 with Dragen (v4.2.4), samtools and bedtools, with quality filtering and exclusion of ENCODE blacklisted regions. Peaks were called with SEACR v1.3 in stringent mode using the IgG controls for background subtraction. Peaks were annotated with HOMER (v4.11.1).

Molecular Dynamics Simulation
MD simulations were conducted with AmberToolsv25 and Amber 24 using the ff19SB force field. Systems were equilibrated and simulated under NPT conditions with no constraints. Trajectory visualization was performed in VMD (v1.9.4). NAMD v2.14 was used for pairwise energy calculations.

Statistical Analysis
Statistical analyses were performed using GraphPad Prism 10, R v4.4.2, Perseus v2.0.11.0, and HOMER v4.11.1. Fisher's exact test was used to assess concordance between RNA-seq and ATAC-seq results. Mass spectrometry data were analyzed in Perseus v2.0.11.0 using unpaired two-tailed t-tests on log-transformed LFQ intensities. Cell proliferation data were compared using unpaired two-tailed t-tests. No multiple testing correction was applied for mass spectrometry or cell proliferation analyses. Motif enrichment was assessed using HOMER v4.11.1 with a one-sided binomial test against a dinucleotide-shuffled background. Differential chromatin accessibility and gene expression were determined using DESeq2. Benjamini-Hochberg correction was applied for multiple comparisons in motif enrichment, differential accessibility, and differential expression analyses. For molecular dynamics simulations, the statistical significance of pairwise energy interactions was assessed using two-tailed one-sample t-tests across 12 independent replicates against a null hypothesis mean of zero, with no correction for multiple comparisons. All biological experiments were performed in at least two independent experiments with two technical replicates. Exact parameters for each statistical test are described in the relevant figure legends and Methods sections.

For manuscripts utilizing custom algorithms or software that are central to the research but not yet described in published literature, software must be made available to editors and reviewers. We strongly encourage code deposition in a community repository (e.g. GitHub). See the Nature Portfolio guidelines for submitting code & software for further information.

Data

Policy information about availability of data

All manuscripts must include a data availability statement. This statement should provide the following information, where applicable:

- Accession codes, unique identifiers, or web links for publicly available datasets
- A description of any restrictions on data availability
- For clinical datasets or third party data, please ensure that the statement adheres to our policy

Sequence data have been deposited at dbGAP under project accession phs003896.v1.p1. Mass spectrometry data has been deposited at PRIDE under project accession PXD062226. Plasmids herein can be found at https://www.addgene.org/Andrew_Hong/. The code for analyzing the data and the raw data tables have been deposited in GitHub (https://github.com/ahonglab/SMARCB1_DMS) and archived on Zenodo (https://doi.org/10.5281/zenodo.18716542). The uncropped immunoblotting images are included in Source Data and Supplementary Information. The underlying data of all applicable figures are exhibited in Source Data. All other data is available from the corresponding author upon reasonable request.

Research involving human participants, their data, or biological material

Policy information about studies with human participants or human data. See also policy information about sex, gender (identity/presentation), and sexual orientation and race, ethnicity and racism.

Reporting on sex and gender	NA
Reporting on race, ethnicity, or other socially relevant groupings	NA
Population characteristics	NA
Recruitment	NA
Ethics oversight	NA

Note that full information on the approval of the study protocol must also be provided in the manuscript.

Field-specific reporting

Please select the one below that is the best fit for your research. If you are not sure, read the appropriate sections before making your selection.

- [x] Life sciences
- [ ] Behavioural & social sciences
- [ ] Ecological, evolutionary & environmental sciences

For a reference copy of the document with all sections, see nature.com/documents/nr-reporting-summary-flat.pdf

Life sciences study design

All studies must disclose on these points even when the disclosure is negative.

Sample size	For deep mutational scanning (DMS) analysis, biological duplicates were employed across three SMARCB1-deficient cell lines to ensure the robustness and reproducibility of variant effect measurements. Although no formal power analysis was performed, the use of biological duplicates is consistent with best practices in deep mutational scanning and mutagenesis studies.<br><br>For RNA-seq, ATAC-seq, and CUT&RUN analyses, biological duplicates were selected in accordance with ENCODE recommendations.<br><br>For cell proliferation assays, at least three biological replicates with two technical replicates each were performed to ensure reproducibility and sufficient power for detecting meaningful differences between conditions.<br><br>For LC-MS/MS analysis, biological triplicates were used to ensure robust and reproducible measurement of protein abundance. This approach is standard in proteomics to account for biological variability and technical noise.<br><br>For molecular dynamics simulations, 12 independent replicates were performed for each condition (WT, W281P, I315R, and S299L). Although no formal power analysis was performed, the use of 12 replicates is consistent with common practices in MD simulation studies and provided sufficient power to detect significant differences in pairwise energy interactions and residue contact frequencies.
Data exclusions	No data were excluded from this analysis.
Replication	All attempts at replication were successful. The number of replicates for each experiment is stated in the figure legends.
Randomization	The order in which samples were run in the LC-MS/MS analysis was randomized to minimize potential systematic biases, such as those introduced by instrument drift, calibration changes, or carryover effects. This randomization also reduces the influence of time-related factors on the data, ensuring that any variation in sample processing is evenly distributed across all groups.
Blinding	Blinding was not done since this study requires the investigator to study differences in cell lines.

Reporting for specific materials, systems and methods

We require information from authors about some types of materials, experimental systems and methods used in many studies. Here, indicate whether each material, system or method listed is relevant to your study. If you are not sure if a list item applies to your research, read the appropriate section before selecting a response.

Materials & experimental systems

n/a	Involved in the study
☐	☒ Antibodies
☐	☒ Eukaryotic cell lines
☒	☐ Palaeontology and archaeology
☒	☐ Animals and other organisms
☒	☐ Clinical data
☒	☐ Dual use research of concern
☒	☐ Plants

Methods

n/a	Involved in the study
☒	☐ ChIP-seq
☒	☐ Flow cytometry
☒	☐ MRI-based neuroimaging

Antibodies

Antibodies used

SMARCB1/INI1/SNF5/BAF47 (Mouse Monoclonal, Santa Cruz Biotechnology, sc-166165, 1:1000 WB dilution)
https://www.scbt.com/p/ini1-antibody-a-5

DPF2/BAF45D (Rabbit Monoclonal, Cell Signaling Technology, 71642S, 1:1000 WB Dilution)
https://www.cellsignal.com/products/primary-antibodies/dpf2-baf45d-e7n8j-rabbit-mab/71642

SMARCC2/BAF170 (Rabbit Monoclonal, Cell Signaling Technology, 12760S, 1:1000 WB Dilution)
https://www.cellsignal.com/products/primary-antibodies/smarcc2-baf170-d8o9v-rabbit-mab/12760

SMARCA4/BRG1 (Mouse Monoclonal, Santa Cruz Biotechnology, sc-17796, 1:1000 WB)
https://www.scbt.com/p/brg-1-antibody-g-7

Lamin A/C (Rabbit Monoclonal, Cell Signaling Technology, 2032S, 1:1000 WB)
https://www.cellsignal.com/products/primary-antibodies/lamin-a-c-antibody/2032

β-actin (Rabbit Monoclonal, Cell Signaling Technology, - 8457S, 1:1000 WB dilution)
https://www.cellsignal.com/products/primary-antibodies/b-actin-d6a8-rabbit-mab/8457

For CUT&RUN:
IgG control (Rabbit Polyclonal, EpiCypher, Cat# 13-0042k, 1uL per reaction)
https://www.epicypher.com/product/cutana-igg-negative-control-antibody-for-cutrun-and-cuttag/

SMARCA4 (Rabbit Monoclonal, Cell Signaling Technologies, D1Q7F, 1uL per reaction)
https://www.cellsignal.com/products/primary-antibodies/brg1-d1q7f-rabbit-mab/49360

SMARCB1 (Rabbit Monoclonal, Cell Signaling Technologies, D8M1X, 1uL per reaction)
https://www.cellsignal.com/products/primary-antibodies/smarcb1-baf47-d8m1x-rabbit-mab/91735

SMARCE1 (Rabbit Monoclonal, Cell Signaling Technologies, E6H5J, 1uL per reaction)
https://www.cellsignal.com/products/primary-antibodies/smarce1-baf57-e6h5j-rabbit-mab/33360

Validation

All antibodies are commercially available and validation information can be found at manufacturer's website (see above).

Eukaryotic cell lines

Policy information about cell lines and Sex and Gender in Research

Cell line source(s)

G401 (ATCC-CRL1441). BT16 cells were from Charles David James, Northwestern University. CCLF_PEDS9001_T1 cells were from our prior publication where we established the cell line models.

Authentication

Whole genome sequencing (WGS) were performed on all samples.

Mycoplasma contamination

Cell lines were regularly tested for mycoplasma contamination using the Lonza Mycoplasma kit.

Commonly misidentified lines
(See ICLAC register)

No commonly misidentified cell lines were used in this study.

Plants

Seed stocks	NA
Novel plant genotypes	NA
Authentication	NA
