# Supplementary material for: SMARCB1 missense mutants disrupt SWI/SNF complex stability and remodeling activity
Source: Nat Commun. 2026 Apr 8;17:4987. doi: 10.1038/s41467-026-71531-8 (PMC13237135; doi:10.1038/s41467-026-71531-8)
Supplement: Supplementary file 4 — Source Data [file 41467_2026_71531_MOESM4_ESM.zip › Source Data/Description of Additional Supplementary Files.pdf]

Description of Additional Supplementary Files

Files included in Source Data

Supp Data 1 – Figure 1

- Fig. 1A: Raw data plotted in Fig. 1A
- Fig. 1A – VAF: Allelic frequency data from GENIE for variants presented in Fig. 1A
- Fig 1B: Raw CADD data for data plotted in Fig. 1B.
- Fig. 1C: Mutation data for cell lines used in study
- Fig. 1D: Quality control for deep mutational screen experiments
- Fig. 1E: Analyzed functional scores from deep mutational scanning experiments
- Fig. 1F: Concordance between CADD and DMS data
- Fig. 1G: Raw plot data plotted for ClinVar variants reported in *BRCA1*, *TP53*, *PTEN*, and *SMARCB1*
- Fig. 1G – Source Data: Underlying source data used to derive Fig. 1G

Supp Data 2 – Figure 2

- Fig. 2A,B: Analyzed functional scores used to generate plots in Fig. 2A and Fig. 2B
- Fig. 2C: Missense mutations that reached a z-score > 2 from DMS experiments
- Fig. 2D: Amino acid resolution of residue intolerance for each residue > 2SD above mean
- Fig. 2F: Population missense variants observed in GnomAD v4.1.0 in *SMARCB1* RPT2 domain
- Fig. 2G: Uncropped immunoblots for Fig. 2G
- Fig. 2I: Raw cell proliferation data for RPT2 variants tested

Supp Data 3 – Figure 3

- Fig. 3A: Uncropped immunoblots presented in Fig 3A
- Fig. 3B – W281: Analysis of SMARCA4 MS-IP data for W281P vs WT comparison
- Fig. 3B – I315: Analysis of SMARCA4 MS-IP data for I315R vs I315I comparison
- Fig. 3C: Unique peptide counts of dissociated SWI/SNF subunits
- Fig. 3E: Uncropped immunoblots presented in Fig. 3E
- Fig. 3F: Quantification of SMARCA4 fractions from immunoblots presented in Fig. 3E and Supplementary Fig. 6G

Supp Data 4 – Figure 4

- Fig. 4A: RPT cleft VDW energy analysis
- Fig. 4B – W281P: Contact frequency analysis for W281P
- Fig. 4C – I315R: Contact frequency analysis for W281P
- Fig. 4C: Residue level VDW and Elec energy analysis

Supp Data 5 – Figure 5

- Fig. 5A: Genomic regions and annotation for all peaks in the context of wild type SMARCB1 re-expression

- Fig. 5B: Genomic annotations for ATAC-seq categories presented in Fig. 5A
- Fig. 5C: Motif enrichment analysis for ATAC-seq categories
- Fig. 5D: Genomic regions from integrated ATAC-seq and CUT&RUN that are both accessible and bound by SWI/SNF in the context of wild type SMARCB1 re-expression
- Fig. 5E: Genomic annotations for genomic regions presented in Fig. 5D
- Fig. 5G: Number of differentially expressed genes from each comparison of bulk RNA-seq analysis
- Fig. 5H – WTvW281X: Concordance analysis for integrated ATAC-seq and RNA-seq analysis for WT vs W281* comparison
- Fig. 5H – W281PvW281X: Concordance analysis for integrated ATAC-seq and RNA-seq analysis for W281P vs W281* comparison
- Fig. 5H – I315IvI315X: Concordance analysis for integrated ATAC-seq and RNA-seq analysis for I315I vs I315* comparison
- Fig. 5H – I315RvI315X: Concordance analysis for integrated ATAC-seq and RNA-seq analysis for I315R vs I315* comparison

Supp Data 6 – Supplementary_Figure 1

- Supp. Fig. 1A: Immunoblot images presented in Supp. Fig. 1A
- Supp. Fig. 1B: Raw cell proliferation data for WT and W281* variants
- Supp. Fig. 1D: Immunoblot images presented in Supp. Fig. 1D
- Supp. Fig 1E: Raw cell proliferation data for R377R, R377H, and R377* variants
- Supp. Fig. 1G: Raw CADD data for reported SMARCB1 SNVs
- Supp. Fig. 1H,J: Raw AlphaMissense data for SMARCB1 substitutions
- Supp. Fig. 1I,K: Raw REVEL data for reported SMARCB1 SNVs.
- Supp. Fig. 1L: cDNA overexpression screen raw data
- Supp. Fig. 1M: Immunoblot images presented in Supp. Fig. 1M
- Supp. Fig. 1N: Immunoblot of images presented in Supp. Fig. 1N
- Supp. Fig. 1O: Detection threshold raw data from DMS screen

Supp Data 7 – Supplementary_Figure 2

- Supp. Fig. 2A: Analyzed DMS datasets used to generate cell line level data
- Supp. Fig 2B,C: ClinVar-annotated SMARCB1 missense variants with corresponding functional score
- Supp. Fig. 2D: Functional scores ordered by most deleterious amino acid type across all residues
- Supp. Fig. 2E: Individual amino acid substitution scores for all intolerant residues
- Supp. Fig. 2F: Per-residue amino acid selectivity and mean functional effect for all SMARCB1 residues with structural annotations derived from AlphaFold predictions

Supp Data 8 – Supplementary_Figure 4

- Supp. Fig. 4: Population missense variants observed in GnomAD v4.1.0 in *SMARCB1*

Supp Data 9 – Supplementary_Figure 5

- Supp. Fig. 5B: Patient mutational frequency observed in AACR GENIEv16 and corresponding functional scores
- Supp. Fig. 5C: Immunoblot of images presented in Supp. Fig. 5C
- Supp. Fig 5E: Raw cell proliferation data for WT, W281P, and W281* variants

Supp Data 10 – Supplementary_Figure 6

- Supp. Fig. 6B: Raw immunoblot quantification values from those presented in Fig. 3A
- Supp. Fig. 6C: Fold change values from SWI/SNF subunits presented in Fig. 3B
- Supp. Fig. 6D: Immunoblot of images presented in Supp. Fig. 6D
- Supp. Fig. 6E: WTvW281X: Analysis of SMARCA4 MS-IP data for W281* vs WT comparison
- Supp. Fig. 6E: I315IvI315X: Analysis of SMARCA4 MS-IP data for I315* vs I315I comparison
- Supp. Fig. 6F: SWI/SNF dissociation values across all comparisons
- Supp. Fig. 6G: Immunoblot of images presented in Supp. Fig. 6G

Supp Data 11 – Supplementary_Figure 7

- Supp. Fig. 7A: Aggregate motif density analysis raw data
- Supp. Fig. 7B: Normalized SMARCB1 transcript levels assessed by bulk RNA-seq
- Supp. Fig. 7C – WTvW281X: Differential expression analysis from WT vs W281* comparison
- Supp. Fig. 7C – W281PvW281X: Differential expression analysis from W281P vs W281* comparison
- Supp. Fig. 7C – I315IvI315X: Differential expression analysis from I315I vs I315* comparison
- Supp. Fig. 7C – I315RvI315X: Differential expression analysis from I315R vs I315* comparison
- Supp. Fig. 7D: Normalized transcript counts for top 25 most differentially expression genes upon functional SMARCB1 induction
